# Association Between Preadmission Metformin Use and Outcomes in Intensive Care Unit Patients With Sepsis and Type 2 Diabetes: A Cohort Study

**DOI:** 10.3389/fmed.2021.640785

**Published:** 2021-03-29

**Authors:** Qilin Yang, Jiezhao Zheng, Weiyan Chen, Xiaohua Chen, Deliang Wen, Weixiao Chen, Xuming Xiong, Zhenhui Zhang

**Affiliations:** Department of Critical Care, The Second Affiliated Hospital of Guangzhou Medical University, Guangzhou, China

**Keywords:** metformin, mortality, sepsis, type 2 diabetes, PSM

## Abstract

**Background:** Sepsis is a deadly disease worldwide. Effective treatment strategy of sepsis remains limited. There still was a controversial about association between preadmission metformin use and mortality in sepsis patients with diabetes. We aimed to assess sepsis-related mortality in patients with type 2 diabetes (T2DM) who were preadmission metformin and non-metformin users.

**Methods:** The patients with sepsis and T2DM were included from Medical Information Mart for Intensive Care -III database. Outcome was 30-day mortality. We used multivariable Cox regression analyses to calculate adjusted hazard ratio (HR) with 95% CI.

**Results:** We included 2,383 sepsis patients with T2DM (476 and 1,907 patients were preadmission metformin and non-metformin uses) between 2001 and 2012. The overall 30-day mortality was 20.1% (480/2,383); it was 21.9% (418/1,907), and 13.0% (62/476) for non-metformin and metformin users, respectively. After adjusted for potential confounders, we found that preadmission metformin use was associated with 39% lower of 30-day mortality (HR = 0.61, 95% CI: 0.46–0.81, *p* = 0.007). In sensitivity analyses, subgroups analyses, and propensity score matching, the results remain stable.

**Conclusions:** Preadmission metformin use may be associated with reduced risk-adjusted mortality in patients with sepsis and T2DM. It is worthy to further investigate this association.

## Background

Sepsis, caused by a dysregulated host response to infection, is a life-threatening organ dysfunction (1). Although the treatment of sepsis has developed rapidly in the past few years, sepsis incidence and mortality are still climbing. Conservative estimates indicate that sepsis is a leading cause of mortality and critical illness worldwide (1–3). To date, the exact mechanism remains unclear, but it is widely postulated that the release of inflammatory factors by innate immune cells plays an important role in the pathogenesis of sepsis (4, 5).

Metformin has become the most common and first-line biguanide antihyperglycemic agent (6) and because of its anti-inflammatory properties, such as anti-oxidant and anti-inflammatory properties (7), which is associated with lower all-cause mortality compared with other hypoglycemic (8). Several studies demonstrated that there was an association between preadmission metformin use and reduced mortality in patients with sepsis (9, 10). Others reported this relationship did not exist (11, 12). Since these results are still controversial, a large cohort study is needed to confirm the association between preadmission metformin use and mortality in patients with sepsis and type 2 diabetes (T2DM).

## Methods

We enrolled patients with sepsis and T2DM who were exposed or not exposed to preadmission metformin in the database of Medical Information Mart for Intensive Care (MIMIC)-III (version 1.4). More than 60,000 patients who stayed in intensive care unit (ICU) of Beth Israel Deaconess Medical Center between 2001 and 2012 were comprised in this real-world and freely-available MIMIC-III database (13). One author Qilin Yang obtained approval to exploit the database (certification number 7634793). All reporting followed the Strengthening the Reporting of Observational Studies in Epidemiology guidelines (14).

### Study Population

Patients with sepsis and T2DM were eligible for our study. Sepsis was defined as an infection combined with evidence of organ dysfunction based on the third sepsis definition (1). Organ dysfunction was represented by an increased sequential organ failure assessment (SOFA) score of two points or more (1, 4). The diagnosis of infection was considered according to the International Classification of Disease, Ninth Revision (ICD-9) categorized by Argus et al. (15). Septic shock was defined as sepsis patients with vasopressor usage (16). We assumed a baseline SOFA of zero for all patients (4). The diagnosis of diabetes was also based on ICD-9. Only adult patients (age >16 years) were included. For patients with recurrent ICU admissions, only the first ICU admission was considered (17). We excluded patients with type 1 diabetes who do not have a clear indication for metformin. We also excluded patients diagnosed with chronic renal failure based on ICD-9, which is a relative contraindication to metformin therapy before 2014 (18).

### Metformin Use

Preadmission metformin use was defined as a record of using metformin in “Medications on admission” in MIMIC-III.

### Covariates

We used the same set of prespecified covariates, which was based on the established predictor of sepsis outcomes (16, 19, 20). We included the following variables: heart rate, mean arterial pressure (MAP), respiratory rate, SPO_2_, white blood cell (WBC) count, hemoglobin, platelet, creatinine, glucose, simplified acute physiology score (SAPS) II score, ventilator use, vasopressor use, renal replace treatment (RRT) use, and comorbidity disease included cardiovascular disease, liver disease, malignancy, neurological disease, chronic pulmonary disease, hypertension, glycated hemoglobin (HBA_1_C), use of statin, use of insulin and use of aspirin before admission. Vasopressor included norepinephrine, epinephrine, phenylephrine, vasopressin, dopamine, dobutamine, and isoprenaline. Basic information for hospital admission registration which contained demographic characteristics, marital status, insurance, admission type, service unit, and admission time was also extracted. These variables included those representing the health habits of patients who received preadmission metformin that may capture a healthy user effect (21).

### Outcome

The outcome was 30-day mortality.

### Statistical Analysis

Multivariable Cox regression analyses were adopted to assess the independent association between preadmission metformin use and 30-day mortality. An extended Cox model approach was used for different covariates adjusted models (22). Survival curves were plotted by Kaplan–Meier and log-rank analyses. Subgroup analyses were stratified by some relevant effect covariates.

Descriptive analysis was applied to all participants. Categorical variables were expressed as proportions (%). Continuous data were expressed as mean and standard deviation (SD) or median and interquartile range (IQR), as appropriate. Variables were compared using the chi-square tests (categorical variables) and One-Way ANOVA (normal distribution), Kruskal-Wallis (skewed distribution) test, respectively.

All the analyses were performed with the statistical software packages R 3.3.2 (http://www.R-project.org, The R Foundation) and Free Statistics software versions 1.1. A two-tailed test was performed and *p* < 0.05was considered statistically significant.

### Sensitivity Analyses

Previous studies reported that elimination half-life of metformin during multiple dosages in healthy patients was 5 h (23, 24). We exclude patients who stayed in the general ward for more than 5 h for sensitivity analyses.

To robust of our findings, we performed a propensity score matching (PSM). A 1:1 nearest neighbor matching algorithm was applied and a caliper width was 0.01. A multivariable logistic regression model was used among those who did and did not have preadmission metformin usage (22, 24). The variables selected to generate the propensity score were as follows: age, sex, ethnicity, marital status, insurance, admission type, service unit, heart rate, MAP, respiratory rate, SPO_2_, WBC, serum creatinine, hemoglobin, platelet, ventilator use, vasopressor use, SAPS II score, HBA_1_C, use of statin, use of insulin, and use of aspirin before admission. The PSM degree was estimated by a standardized mean difference. A threshold < 0.1 was considered acceptable (25). It was indispensable to calculate the hazard ratio (HR) for 30-day mortality, a univariable Cox proportional hazards regression model with the robust variance estimator was applicable.

## Results

### Population

**Figure 1:**
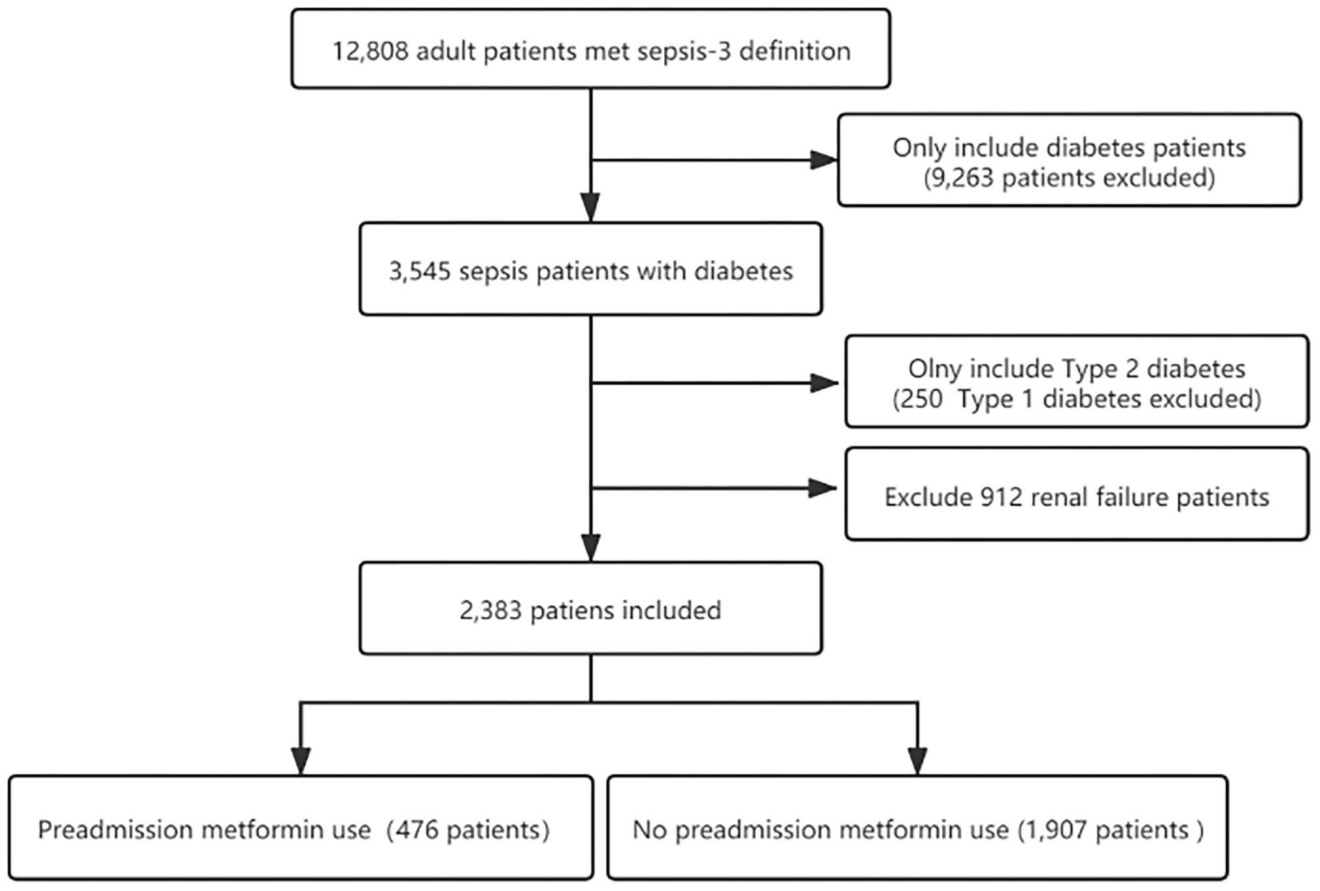
The flow chart of the study.

Three thousand five hundred and forty five individuals with diabetes who underwent sepsis were identified according to the sepsis-3 criterion. After excluding type 1 diabetes and renal failure patients, the final cohort included 2,383 patients with sepsis and T2DM. Of these patients, 476 (20%) were preadmission metformin users. The flow chart of the study patients selection is presented in Figure 1.

### Baseline Characteristics

The baseline characteristics of all participants were listed in Table 1. The age of all participation was 70.2 ± 13.1, 48% was female, 1,649 (69.2%) were white individuals, and 734 (30.8%) were non-white individuals. In preadmission metformin use group, more individuals had private insurance [399 (20.9%) VS. 125 (26.3%)], electively admitted to ICU [136 (7.1%) VS. 47 (9.9%)] and less individuals had congestive heart failure [536 (28.1%) VS. 92 (19.3%)]. The overall 30-day mortality was 20.1% (480/2,383). The 30-day mortality for non-metformin and metformin users was 21.9% (418/1,907) and 13.0% (62/476), respectively.

**Table 1:** Baseline characteristics of participants.

**Covariates**	**All patients**	**Preadmission metformin use**		***P*-value**
	**(*n =* 2,383)**	**No (*n =* 1,907)**	**Yes (*n =* 476)**	
Age(years)	70.2 ± 13.1	69.5 ± 12.6	70.1 ± 12.4	0.263
Female, sex, n (%)	1,145 (48.0)	915 (48.0)	230 (48.3)	0.895
Ethnicity, white, n (%)	1,649 (69.2)	1,303 (68.3)	346 (72.7)	0.065
Marital status, n (%)				0.212
Single/divorced	658 (27.6)	525 (27.5)	133 (27.9)	
Married	1,109 (46.5)	871 (45.7)	238 (50.0)	
Other	616 (25.8)	511 (26.8)	105 (22.1)	
Insurance, n (%)				0.013
Medicaid	1,810 (76.0)	1,464 (76.8)	346 (72.7)	
Private	524 (22.0)	399 (20.9)	125 (26.3)	
Other	49 (2.1)	44 (2.3)	5 (1.1)	
Admission type, n (%)				0.044
Elective	183 (7.7)	136 (7.1)	47 (9.9)	
Emergency	2,200 (92.3)	1,771 (92.9)	429 (90.1)	
Service unit, n (%)				0.166
CCU	332 (13.9)	266 (13.9)	66 (13.9)	
CSRU	288 (12.1)	217 (11.4)	71 (14.9)	
MICU	1,157 (48.6)	937 (49.1)	220 (46.2)	
SICU	384 (16.1)	315 (16.5)	69 (14.5)	
TSICU	222 (9.3)	172 (9.0)	50 (10.5)	
Heart rate (bpm)	87.1 ± 16.1	86.9 ± 16.2	88.2 ± 16.0	0.109
MAP (mmHg)	75.7 ± 10.5	75.8 ± 10.6	75.3 ± 10.0	0.405
Respiratory rate (bpm)	20.0 ± 4.3	19.7 ± 4.2	20.4 ± 4.2	0.128
SPO_2_ (%)	96.9 ± 2.7	96.9 ± 2.8	96.9 ± 2.0	0.935
Glucose (mg/dL)	165.8 ± 54.2	165.2 ± 54.8	168.4 ± 52.0	0.245
WBC (×10^9^)	13.7 (9.9–18.7)	13.7 (9.9–18.8)	13.6 (9.9–18.4)	0.822
SCr (mg/dL)	1.2 (0.9–1.8)	1.3 (0.9–1.9)	1.2 (0.9–1.7)	0.488
Hemoglobin (g/L)	10.0 ± 2.1	9.8 ± 2.0	9.9 ± 2.0	0.240
Platelet (×10^12^)	188.0 (127.0–257.0)	188.0 (125.0–260.0)	187.0 (137.0–246.2)	0.764
Lactate (mmol/L)	2.4 (1.6–4.0)	2.4 (1.5–4.0)	2.4 (1.6–3.9)	0.676
HBA1C (%)	7.2 ± 2.0	7.1 ± 2.0	7.3 ± 1.7	0.007
Tested HBA1C, n(%)	780 (32.7%)	610 (32.0%)	170 (35.7%)	0.121
SAPS II score	41.6 ± 14.1	41.9 ± 14.2	40.5 ± 13.4	0.057
Infection site				
Respiratory system	602 (25.4%)	493 (26.0%)	109 (22.9%)	0.326
Cardiovascular system	808 (34.1%)	641 (33.8%)	167 (35.2%)	
Digestive system	185 (7.8%)	149 (7.9%)	36 (7.6%)	
Urogenital system	443 (18.7%)	341 (18.0%)	102 (21.5%)	
Other	331 (14.0%)	270 (14.3%)	61 (12.8%)	
Preadmission medications				
Statin	945 (39.7%)	658 (34.5%)	287 (60.3%)	<0.001
Insulin	629 (26.4%)	539 (28.3%)	90 (18.9%)	<0.001
Aspirin	551 (23.1%)	423 (22.2%)	128 (26.9%)	0.029
Ventilator use, n (%)	1,205 (50.6)	1,007 (52.8)	259 (54.4)	0.530
Vasopressor use, n (%)	1,037 (43.5)	832 (43.6)	205 (43.1)	0.825
RRT, n (%)	78 (3.3)	65 (3.4)	13 (2.7)	0.457
Comorbidity disease, n (%)				
Congestive heart failure	628 (26.4)	536 (28.1)	92 (19.3)	<0.001
Liver disease	220 (9.2)	189 (9.9)	31 (6.5)	0.220
Malignancy	228 (9.6)	176 (9.2)	52 (10.9)	0.261
Neurological disease	308 (12.9)	259 (13.6)	49 (10.3)	0.056
Chronic pulmonary disease	573 (24.0)	462 (24.2)	111 (23.3)	0.679
Hypertension	41 (1.7)	35 (1.8)	6 (1.3)	0.533
30-day mortality, n (%)	480 (20.1)	418 (21.9)	62 (13.0)	<0.001

*Bpm, beat per minute; CCU, coronary care unit; CSRU, cardiac surgery recovery unit; LOS, length of stay; MICU, medical intensive care unit; MAP, mean arterial pressure; RRT, renal replace treatment; SICU, surgical intensive care unit; SAPS, simplified acute physiology score; TSICU, trauma and surgical intensive care unit; WBC, white blood count; SCr, serum creatinine; HBA1C, glycated hemoglobin*.

### Relationship Between Preadmission Metformin Usage and 30-Day Mortality

**Figure 2:**
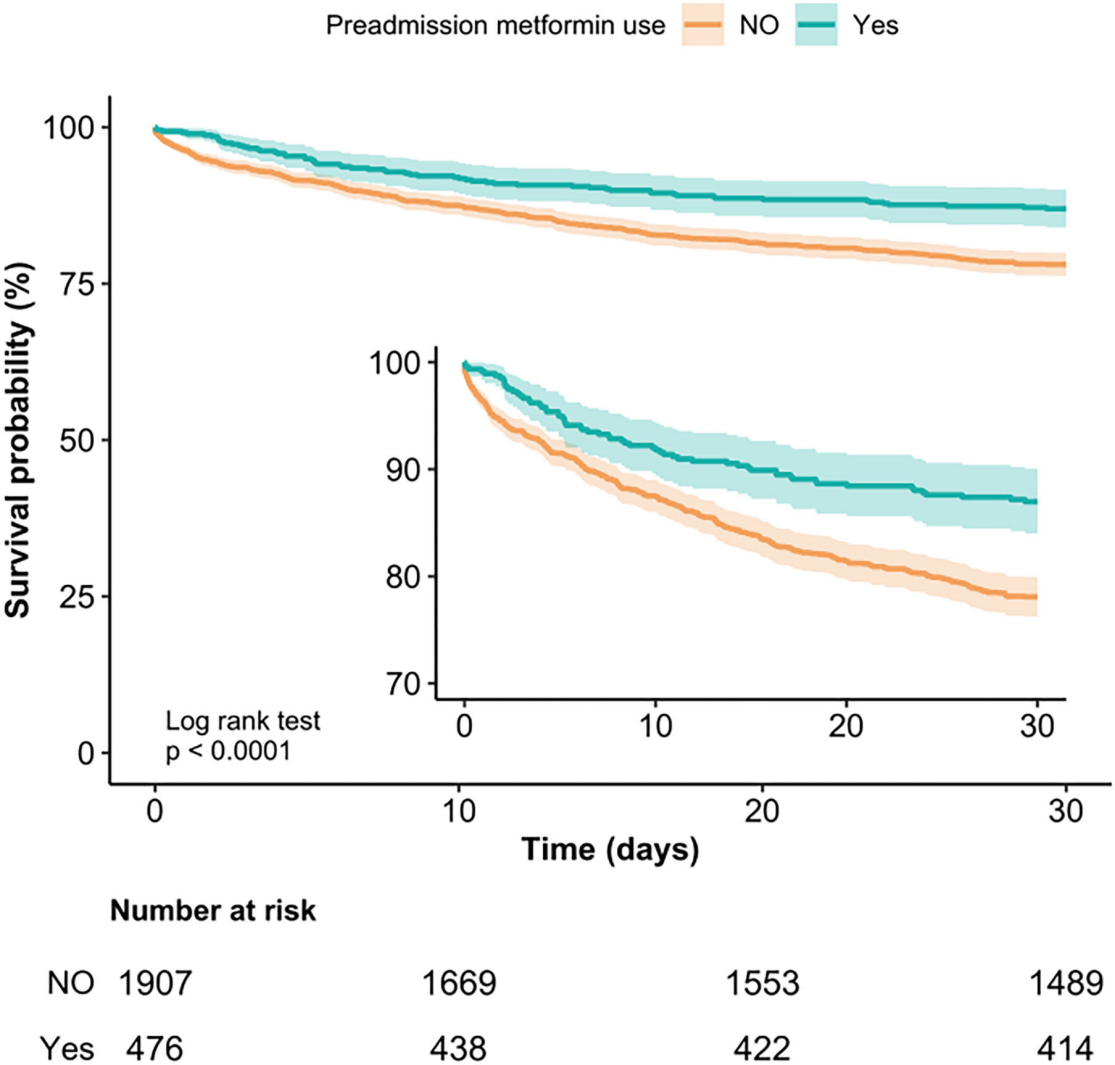
Kaplan-Meier Survival Curves for day 30 of sepsis patients with type 2 diabetes.

**Figure 3:**
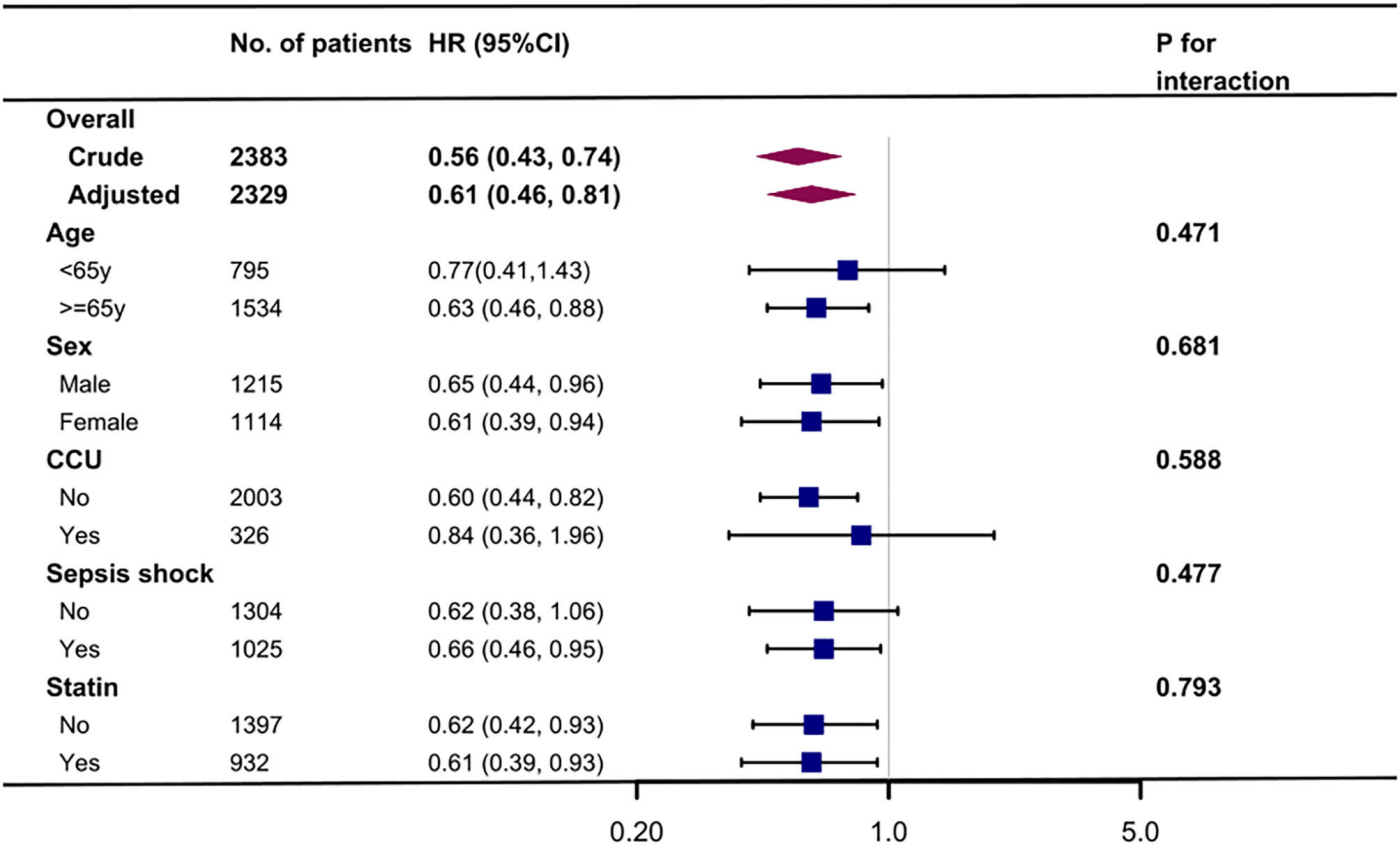
Association between preadmission metformin use and 30-day mortality according to baseline characteristics. Each stratification adjusted for all the factors (age, sex, ethnicity, marital status, insurance, admission type, service unit, heart rate, MAP, respiratory rate, SPO_2_, WBC, SCr, hemoglobin, platelet, ventilator use, vasopressor use, comorbidity disease, and SAPS II) except the stratification factor itself. CCU, coronary care unit.

Kaplan-Meier curve showed there was lower mortality by day 30 in patients with preadmission metformin use (Log-rank test: *p* < 0.0001, Figure 2). In the extended multivariable Cox models (Table 2), we observed that the hazard ratios (HRs) of preadmission metformin use were consistently significant in all five models (HRs range 0.56–0.61, *p* < 0.05 for all). After adjustment for all covariates in Table 1, a 39% lower of 30-day mortality could be shown in patients with preadmission metformin use (HR = 0.61, 95% CI: 0.46–0.81, *p* = 0.007, model 5, Table 2, Figure 3). Although subgroup analysis was performed according to the confounders including age, sex, SAPS II score, vasopressor use, and comorbidity diseases (Figure 3), we did not observe any significant interaction in the subgroups (*p-*value for interaction > 0.05 for all).

**Table 2:** Association between preadmission metformin use and 30-day mortality using an extended model approach.

	***N***	**Hazard ratio of preadmission metformin**	**95% confidence interval**	***P*-value**
Model 1	2,383	0.56	(0.43, 0.74)	<0.001
Model 2	2,383	0.58	(0.44, 0.75)	<0.001
Model 3	2,354	0.55	(0.42, 0.73)	<0.001
Model 4	2,329	0.55	(0.41, 0.72)	<0.001
Model 5	2,329	0.61	(0.46, 0.81)	0.007

Adjusted covariates: Model 1 = preadmission metformin use.

Model 2 = Model 1+ (age, sex, ethnicity).

Model 3 = Model 2+ (marital status, insurance, admission type, service unit, heart rate, MAP, respiratory rate, SPO_2_, WBC, creatinine, hemoglobin, platelet, ventilator use, vasopressor use, comorbidity diseases, SAPS II).

Model 4 = Model 3+ (glucose, RRT use, tested HBA_1_C, infection site, insulin, aspirin).

*Model 5 = Model 4+statin*.

### Sensitive Analysis

After excluding patients stay in the general ward for more than 5 h [the median stay time was 0.02 (0.02–0.03) h], there were 1,589 patents left, and the relationship between preadmission metformin usage and in-hospital mortality stay reliable (HR = 0.52, 95% CI: 0.35–0.77, *p* = 0.001). However, there were 794 patients stay in the general ward for more than 5 h [the median stay time was 2.40 (0.86–5.10) days], and we found a negative result in this group (HR = 0.83, 95% CI: 0.45–1.53, *p* = 0.551). However, there are no differences in two groups (p for interaction is 0.46).

After PSM, 455 pairs of each group were well-matched (Supplementary Table 1). There are no significant differences between the two matched groups. Among the 455 propensity-matched pairs, the 30-day mortality was significantly lower in the preadmission metformin use group [57 (12.5) vs. 85 (18.7), *p* = 0.014]. The hazard ratio (HR) for 30-day mortality was 0.56 (95% CI: 0.42–0.73, *p* < 0.0001) calculated by the univariable Cox proportional hazards regression model.

## Discussion

To our best knowledge, this study is the largest cohort on association of preadmission metformin use and mortality in sepsis patients with T2DM. In the study, metformin users with sepsis and T2DM had a lower risk-adjusted 30-day mortality in comparison to patients who did not use it. This result remained robust in the comparisons after PSM.

Consistent with our results, previous studies demonstrated that preadmission metformin use was associated with a decrease in in-hospital mortality or 28-day mortality in patients with sepsis and diabetes (9, 10). However, the definition of sepsis of those studies was according to the old version, and conclusions may not be appropriate for the sepsis-3 definition. Our study extended these findings in patients with sepsis-3 definition and T2DM.

A meta-analysis confirmed that preadmission metformin users had a 41% (OR = 0.59, 95% CI: 0.43–0.79) lower mortality than non-user inpatient with sepsis and diabetes (26). Their results are akin to our findings. However, this meta-analysis only enrolled 1,282 patients (26) and still overlooked several important confounders, such as serum glucose levels, differences baselines in the SAPS II (27), and RRT in both arms (28). Our study had a much larger cohort (*n* = 2,383) and used an extended model approach to adjust the potential confounders and found a stable relationship between preadmission metformin use and 30-day mortality.

Jochmans et al. conducted a retrospective cohort study in a French ICU (*n* = 635) and described preadmission metformin did not affect in-hospital mortality (OR = 0.75, 95% CI: 0.44–1.28) (11). In Jochmans' cohort, preadmission metformin use was significantly associated with lower mortality in patients with septic shock after multivariate analysis (OR = 0.61, 95% CI: 0.36–0.99) (11). This phenomenon also can be found in our study, preadmission metformin use was significantly associated with lower 30-day mortality for patients with septic shock (HR = 0.66, 95% CI: 0.46–0.95).

Oh et al. conducted a nationwide sample cohort study in South Korea and found prior metformin therapy was not significantly associated with the risk of sepsis and 30-day mortality after diagnosis of sepsis among diabetes patients (12). Compared with our study, some pivotal risk factors, such as SAPS II score (29) and vasopressor usage (20), were not effectively controlled in the study by Oh et al. (12).

It is still unclear the mechanism of preadmission metformin use associated with lower mortality in patients with sepsis and diabetes. Metformin could not only improve autophagy and mitochondrial function in diabetes (30) but also decrease inflammation by down-regulate pro-inflammatory cytokines, such as IL-6 and TNF-α (7, 31). Moreover, metformin may play a potential role in antimicrobial therapy. Laboratory tests had shown that metformin was effective against multiple pathogens, including Staphylococcus aureus, Pseudomonas aeruginosa, Trichinella spiralis, hepatitis B virus, hepatitis C virus, and human immunodeficiency virus (32). So the impact on sepsis resulted from the antimicrobial effect which metformin performed may be beneficial.

This study has several noteworthy limitations. First, residual confounders such as duration of type 2 diabetes mellitus, smoking status and alcohol use potentially exist, as with all retrospective analyses. We adjusted for possible confounders and minimized the influence of factors that may lead to outcome bias through the PSM. Second, as the study population only contains patients with sepsis and type 2 diabetes, and they did not suffer from renal failure, it may not be generalizable to patients with sepsis and type 1 diabetes or renal failure. Third, one of the contraindications to metformin was myocardial infarction during the previous month, but we were unable to exclude these patients (11). We exclude patients admitted to the CCU as a proxy. The result was still robust and reliable. Fourth, it was likely to be more prone to unrecorded for the record of metformin in “Medications on admission” in this study. The preadmission metformin use in patients with sepsis and diabetes was lower than previously reported (33). However, it is noteworthy that the bias of potential exposure misclassification resulting from such errors would toward the null, leading to an underestimation of the association between preadmission metformin use and in-hospital mortality. Finally, the causes of death were not recorded in the MIMIC-III database, we could not conduct a competing risk analysis.

## Conclusions

Preadmission metformin use may be associated with reduced risk-adjusted mortality in patients with sepsis and type 2 diabetes. Further clinical trials are required to confirm and validate this association.

## Data Availability Statement

The raw data supporting the conclusions of this article will be made available by the authors, without undue reservation.

## Ethics Statement

The studies involving human participants were reviewed and approved by The Massachusetts Institute of Technology and Beth Israel Deaconess Medical Center. Written informed consent to participate in this study was provided by the participants' legal guardian/next of kin.

## Author Contributions

QY conducted data analysis and wrote the manuscript. JZ conducted data analysis and modified the manuscript. WC and XC conducted data collection. DW conducted data collection and data interpretation. WC drew the figure. XX and ZZ designed the study and reviewed the manuscript. All authors contributed to the article and approved the submitted version.

## Conflict of Interest

The authors declare that the research was conducted in the absence of any commercial or financial relationships that could be construed as a potential conflict of interest.

## Abbreviations

Bpm: Beat per minute
IQR: Interquartile range
LOS: length of stay
MAP: Mean arterial pressure
MIMIC: Medical Information Mart for Intensive Care
PSM: Propensity score matching
RRT: Renal replace treatment
SD: Standard deviation
SOFA: Sequential organ failure assessment
SAPS: Simplified acute physiology score
TSICU: Trauma and surgical intensive care unit
CCU: Coronary care unit
CSRU: Cardiac surgery recovery unit
ICU: Intensive care unit
MICU: Medical intensive care unit
SICU: Surgical intensive care unit
WBC: White blood cell.
