# Supplementary material for: Association Between Preadmission Metformin Use and Outcomes in Intensive Care Unit Patients With Sepsis and Type 2 Diabetes: A Cohort Study
Source: Front Med (Lausanne). 2021 Mar 29;8:640785. doi: 10.3389/fmed.2021.640785 (PMC8039324; doi:10.3389/fmed.2021.640785)
Supplement: Supplementary file 1 [file Table_1.DOCX]

sTable 1. Baseline characteristics of participants after propensity score matching

| **Covariates** | **Preadmission metformin usage** | | **P value** | **SMD** |
| --- | --- | --- | --- | --- |
|  | No (N=455) | Yes (N=455) |  |  |
| Age(years) | 70.3 ± 12.8 | 69.7 ± 12.7 | 0.428 | 0.053 |
| Female, sex, no.(%) | 231 (50.8) | 234 (51.4) | 0.895 | 0.013 |
| Ethnicity , white, no.(%) | 118 (25.9) | 123 (27) | 0.764 | 0.025 |
| Marital status, no.(%) |  |  | 0.688 |  |
| Married | 213 (46.8) | 220 (48.4) |  | 0.031 |
| Single | 195 (42.9) | 183 (40.2) |  | 0.054 |
| Other | 47 (10.3) | 52 (11.4) |  | 0.035 |
| Insurance,no.(%) |  |  | 0.096 |  |
| Medicaid | 343 (75.4) | 336 (73.8) |  | 0.035 |
| Private | 99 (21.8) | 114 (25.1) |  | 0.078 |
| Other | 13 (2.9) | 5 (1.1) |  | 0.127 |
| Admission type,no.(%) |  |  | 0.820 | 0.023 |
| Elective | 41 (9) | 44 (9.7) |  |  |
| Emergency | 414 (91) | 411 (90.3) |  |  |
| Service unit,no.(%) |  |  | 0.319 |  |
| CCU | 65 (14.3) | 62 (13.6) |  | 0.019 |
| CSRU | 55 (12.1) | 68 (14.9) |  | 0.084 |
| MICU | 223 (49) | 212 (46.6) |  | 0.048 |
| SICU | 75 (16.5) | 63 (13.8) |  | 0.074 |
| TSICU | 37 (8.1) | 50 (11) |  | 0.097 |
| Heart rate (bpm) | 87.8 ± 16.1 | 87.8 ± 15.8 | 0.975 | 0.002 |
| MAP (mmHg) | 75.1 ± 10.2 | 75.3 ± 10.0 | 0.769 | 0.020 |
| Respiratory rate (bpm） | 20.2 ± 4.5 | 20.2 ± 4.0 | 0.736 | 0.022 |
| SPO_2_ (%) | 97.1 ± 2.0 | 96.9 ± 2.0 | 0.228 | 0.080 |
| Glucose (mg/dL) | 165.6 ± 60.3 | 167.8 ± 51.7 | 0.554 | 0.039 |
| WBC (×10^9^） | 15.0 ± 7.4 | 15.3 ± 11.8 | 0.598 | 0.035 |
| Creatinine (mg/dL) | 1.6± 1.2 | 1.6± 1.4 | 0.740 | 0.022 |
| Hemoglobin (g/L) | 10.0 ± 2.1 | 9.9 ± 2.1 | 0.668 | 0.029 |
| Platelet (×10^12^) | 206.7 ± 113.1 | 202.1 ± 102.4 | 0.517 | 0.043 |
| HBA1C（%） | 2.6 ± 3.6 | 2.6 ± 3.6 | 0.798 | 0.017 |
| Tested HBA1C,n（%） | 164 (36) | 165 (36.3) | 1.000 | 0.005 |
| SAPS II score | 40.5 ± 14.1 | 40.7 ± 13.3 | 0.813 | 0.016 |
| Infection site | | | 0.785 |  |
| Respiratory system | 117 (25.8) | 101 (22.2) |  | 0.083 |
| Cardiovascular system | 154 (33.9) | 160 (35.2) |  | 0.028 |
| Digestive system | 29 (6.4) | 33 (7.3) |  | 0.035 |
| Urogenital system | 94 (20.7) | 100 (22) |  | 0.032 |
| Other | 60 (13.2) | 60 (13.2) |  | ＜0.001 |
| Preadmission medications | | | | |
| Statin | 253 (55.6) | 272 (59.8) | 0.227 | 0.085 |
| Insulin | 86 (18.9) | 87 (19.1) | 1.000 | 0.006 |
| Aspirin | 129 (28.4) | 124 (27.3) | 0.767 | 0.025 |
| Ventilator use, n.(%) | 235 (51.6) | 240 (52.7) | 0.791 | 0.022 |
| Vasopressor use, n.(%) | 185 (40.7) | 199 (43.7) | 0.383 | 0.062 |
| RRT, n.(%) | 8 (1.8) | 13 (2.9) | 0.377 | 0.073 |
| Clinical outcomes |  |  |  |  |
| 30-day mortality, n (%) | 85 (18.7) | 57 (12.5) | 0.014 | 0.170 |

Bpm: beat per minute, CCU: coronary care unit, CSRU: cardiac surgery recovery unit, LOS: length of stay, MICU: medical intensive care unit, MAP: mean arterial pressure, RRT: renal replace treatment, SICU: surgical intensive care unit, SAPS: simplified acute physiology score, SOFA: sequential organ failure assessment, TSICU: trauma and surgical intensive care unit, WBC: white blood count.
